# Supplementary material for: Bedside Renal Doppler Ultrasonography and Acute Kidney Injury after TAVR
Source: J Clin Med. 2020 Mar 25;9(4):905. doi: 10.3390/jcm9040905 (PMC7230258; doi:10.3390/jcm9040905)
Supplement: Supplementary file 1 [file jcm-09-00905-s001.pdf]

## Supplementary file: Systemic and renal hemodynamic parameters

### George Ohm’s model to explain the cardio-renal hemodynamic interplay after TAVR?

The well-known Ohm’s law states that V=IxR, where V is voltage, I is current, and R is resistance. Applied to the circulation, the terms are substituted such that P=COxR, where P is pressure, CO is cardiac output (L/min), and R is the vascular resistance in the area of interest. Assuming that the circulatory system is analogous to an electric circuit (Supplementary figure 1), aortic stenosis (AS) patients face 3 major resistors: R1 the total pulmonary resistance made up of left ventricular (LV) filling pressure plus pulmonary vascular resistance that governs LV filling; R2, the resistance offered by the aortic valve; and R3, the systemic vascular resistance (SVR).

Following this principle, if resistance drops without a concomitant increase in output, pressure must also fall. In severe AS, R2 (valve resistance) substantially exceeds R3 (SVR) and TAVR allows an instant and unique reduction of the trans-aortic gradient, normalization of the aortic valve area. R2 drops to normal values (transprosthetic gradient assumed to be 5 to10mmHg). As such, a fall in R2 caused by TAVR may increase LVEF and cardiac output in particular in low-flow low-gradient AS. However Ohm’s model applied to aortic stenosis is too restrictive and doesn’t integrate the dynamic interaction between the heart and the systemic circulation known as the ventriculo-arterial coupling concept.

### The ventriculo-arterial coupling

The dynamic interaction between the heart and the systemic circulation allows the cardiovascular system to provide adequate cardiac output and arterial pressures to ensure adequate organ perfusion. The interplay between cardiac function and arterial system, which in turn affects ventricular performance, is known as the ventriculo-arterial coupling and is affected in AS. Because left ventricular stroke volume depends on myocardial contractility and loading conditions (preload and afterload) both myocardial, valvular, and arterial dysfunction

can lead to ventriculo-arterial decoupling with resulting decrease in stroke volume, cardiac output, and organ perfusion including kidneys.

Addressing the post TAVR AKI question, both systemic and local renal modulation of the arterial system compliance and resistance with respect to left ventricular systolic performance is of paramount importance to address potential cardio-renal interaction The interdependence between the heart and the kidneys in TAVR has been a topic of extensive research but no study to our knowledge was specifically designed to tackle the question whether hemodynamic cardio-renal parameters could predict and influence AKI development.

In this study, we sought to provide extensive data investigating the potential link between cardiac function and output, renal function in a TAVR population with careful cardiac, renal and hemodynamic profiling.

**Echocardiography protocol and hemodynamic parameters**

Concomitant echocardiography was performed after RDU measurements 12 hours prior, 24 hours and 72 hours after TAVR procedure. The following echocardiographic and clinical variables were collected from each patient: heart rate; systolic, diastolic, and mean blood pressures; left ventricular ejection fraction (LVEF); aortic flow time-velocity integral, Aortic velocity time integral and diastolic left ventricular function parameters. Systemic and local renal hemodynamic parameters were calculated according to current definitions and included valvulo-arterial impedance, total arterial load, Pulsatile arterial load assessed by pulse pressure, Systemic arterial compliance, Resistive arterial load, Renal arterial load, Renal pulse pressure, Renal arterial compliance (Supplementary table 1)

**Systemic vascular indices: Rational**

**Valvulo-arterial impedance**

Common indices used for the evaluation of AS severity (aortic valve area, mean transvalvular pressure gradient, peak aortic jet velocity) focus on the extent of aortic valve disease.

However, the ventricle in patients with AS face increased afterload from the valve but also from the arterial system. Reduced systemic aortic compliance increases the afterload and contributes to a reduced left ventricular function (1). Valvulo-arterial impedance incorporates both arterial impedance and valve severity. Valvulo-arterial impedance has been linked in several studies to worse outcome in medically (2)(3) and in TAVR managed aortic stenosis patients (4-8). Valvulo-arterial impedance is calculated by dividing the estimated left ventricular systolic pressure (systolic arterial pressure + mean transvalvular gradient) by the stroke volume indexed for the body surface area.

**Others systemic vascular indices**

Others vascular indices where calculated:

- Total arterial load was measured as indexed arterial elastance ([0.9×systolic blood pressure]/ stroke volume index) (9)(10)

- Pulsatile arterial load was measured by pulse pressure Pulse pressure = Systolic blood pressure – Diastolic blood pressure.

- Systemic arterial compliance, calculated as stroke volume index divided by pulse pressure (11) (12)

- Resistive arterial load was measured by systemic vascular resistance index, which was calculated as ([Diastolic blood pressure +1/3 pulse pressure]×80)/ cardiac index.

The resistive component of arterial load was evaluated by the systemic vascular resistance.The pulsatile load, which is related to the stiffness of the conduit vessels, was appreciated by pulse pressure and systemic arterial compliance

**Renal vascular indices: Rational**

Mean arterial pressure represents the renal preload while renal afterload is considered to be mostly driven by central venous pressure and intraabdominal pressure. While Renal blood flow can be assessed by RDU according to the following formula : [Renal blood flow (mL/min) = time-averaged flow velocity (cm/s) × cross-sectional area mm2 × 60 (s)]; such measurement is known to be difficult to measure with high intra and interobserver variability. An alternative

is to consider that renal blood flow represents 20% of the cardiac output. According to these definitions, local renal hemodynamic criteria can be calculated as follow:

- Total renal arterial load was measured as renal indexed arterial elastance ([0.9× Mean arterial pressure]/ stroke volume index x20%)
- Transrenal pressure was measured by renal pulse pressure Renal pulse pressure = [Mean arterial pressure - (central venous pressure + intraabdominal pressure)]

-Renal arterial compliance calculated as (20%x stroke volume index) divided by renal pulse pressure

**Supplementary table 1: Systemic and renal hemodynamic parameters**

| | Systemic Parameters | Renal Parameters | |
|---|---|---|---|
| Total arterial load | Represents arterial elastance | Total renal arterial load | Represents renal arterial elastance |
| Pulse pressure | Represents pulsatile arterial load | Renal pulse pressure | Represents transrenal pressure |
| Systemic arterial compliance | Represents systemic arterial compliance | Renal arterial compliance | Represents renal arterial compliance |
| Resistive arterial load | Represents systemic vascular resistance | | |
| Valvulo-arterial impedance | Translates both arterial impedance and valve severity | | |
| Resistive arterial load | Represents systemic vascular resistance | | |

**Supplementary figure 1: Circulatory system as an electric circuit**

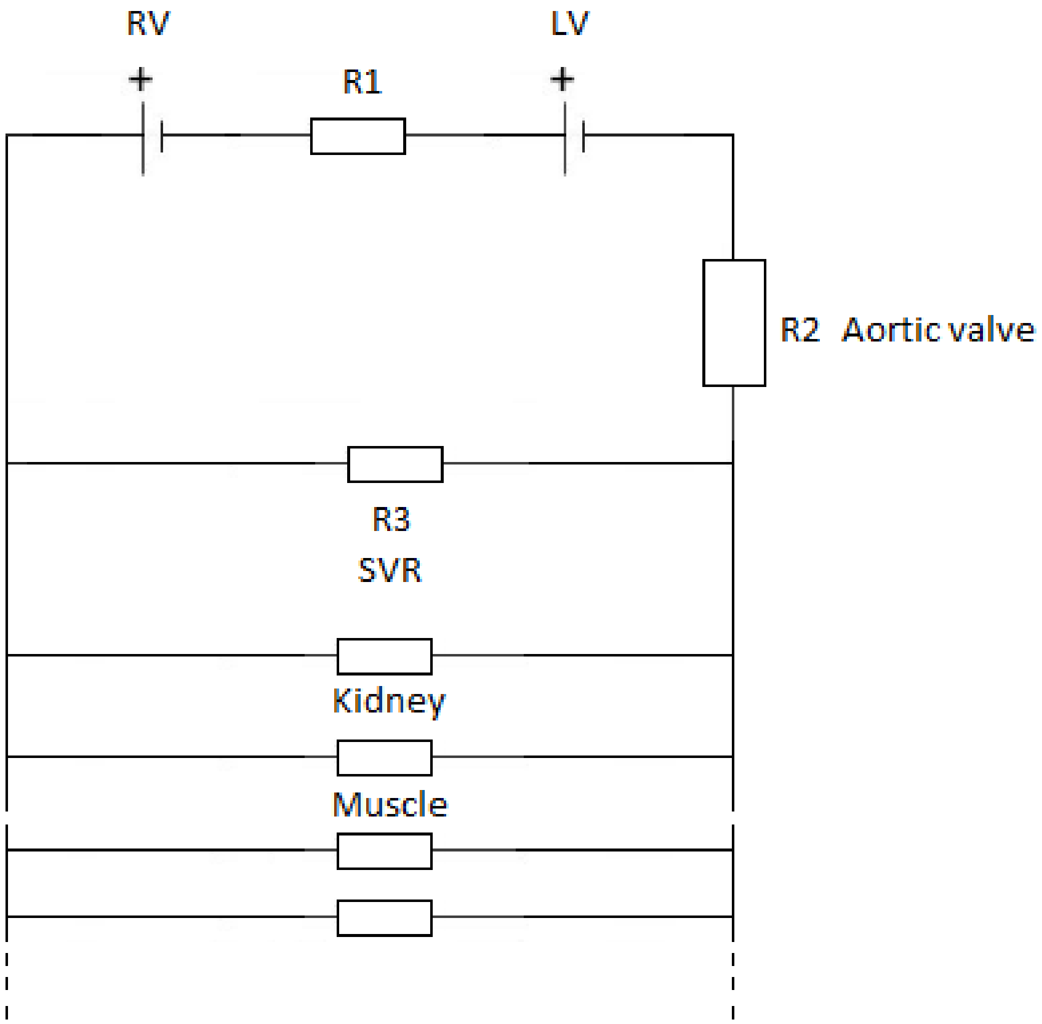


**Figure.** Diagram of the circulation as an electric circuit. Ao, aortic; SVR, systemic vascular resistance; RV, right ventricle; $R_1$, total pulmonary resistance; LV, left ventricle; $R_2$, aortic valve resistance and $R_3$.
